# Propionic Acid Impact on Multiple Sclerosis: Evidence and Challenges

**DOI:** 10.3390/nu16223887

**Published:** 2024-11-14

**Authors:** Lorena Lorefice, Magdalena Zoledziewska

**Affiliations:** 1 Multiple Sclerosis Center, ASL Cagliari, Department of Medical Sciences and Public Health, Binaghi Hospital, University of Cagliari, via Is Guadazzonis 2, 09126 Cagliari, Italy; lorena.lorefice@aslcagliari.it; 2 Institute of Genetic and Biomedical Research (IRGB), Italian National Research Council (CNR), 09042 Monserrato, Italy

**Keywords:** multiple sclerosis, propionic acid, SCFA, gut microbiota, diet

## Abstract

Accumulating evidence suggests that multiple sclerosis (MS) is an environmentally influenced disorder with contributions from life-time exposure to factors including Epstein–Barr virus infection or shifts in microbiome, diet and lifestyle. One suggested factor is a deficiency in propionic acid, a short-chain fatty acid produced by gut bacteria that may contribute to the disease pathology both in animal models and in human cases of MS. Propionate appears to exert beneficial effects on the immune, peripheral and central nervous systems of people with MS (pwMS), showing immunoregulatory, neuroprotective and neurogenerative effects. These functions are crucial, given that MS is characterized by immune-mediated damage of myelin in the central nervous system. Accordingly, propionate supplementation or a modulated increase in its levels through the microbiome and diet may help counteract the pro-inflammatory state in MS by directly regulating immune system and/or by decreasing permeability of gut barrier and blood–brain barrier. This could potentially improve outcomes when used with immune-modulating therapy. However, while its broad effects are promising, further large clinical trials are necessary to evaluate its efficacy and safety in pwMS and clarify its role as a complementary therapeutic strategy. This review provides a comprehensive analysis of the evidence, challenges and limitations concerning propionic acid supplementation in MS.

## 1. Introduction

Multiple sclerosis (MS) is a chronic inflammatory, demyelinating immune-mediated disease of the central nervous system with a complex and multifaceted etiology involving both genetic and environmental factors. Because genetics explains only 30% of the disease risk, the environment is considered the primary contributor. The adulthood onset of MS and its established risk factors further indicate the importance of environmental exposures. Key factors include exposure to the infectious pathogens (Epstein–Barr virus), pollution, smoking, increased population density, lifestyle and diet [1]. In addition, several studies have examined metabolic deficiencies in MS, exploring how they might influence disease susceptibility, progression and severity [2].

The increasing prevalence of MS and other autoimmune disorders can be partially attributed to changes in these environmental factors—and, in particular, dietary habits. The Western-type diet, characterized by excessive intake of fats, and being high in protein, salt, and preservatives, along with reduced intake of plant-derived foods (the source of fiber) is suspected to contribute to MS development and its evolution. Indeed, excessive fat consumption, and consequential obesity—especially in adulthood—are specifically implicated as having a role in the etiology of MS [3]. As one possible mediating mechanism, diet is a major regulator of the composition of the gut microbial ecosystem, with emerging evidence of a bidirectional relationship between gut and the nervous and immune systems. The gut microbiome produces metabolites, including short chain fatty acids (SCFAs), that regulate systemic immune disequilibrium (immunometabolism) and thereby affect autoimmune diseases [4]. Studies in the experimental autoimmune encephalomyelitis (EAE) animal model of MS, as well as human MS, suggest that the gut microbiome mediates autoimmune attacks on the brain, while the brain also regulates gut function in what has been termed the gut–brain axis [5,6,7,8]. The gut–brain axis involves the network of neurons in the intestinal mucosa that regulates digestion. This system is influenced by secreted immune effector molecules [9]. SCFAs in the gut could regulate neuronal activity [10,11]. In addition, SCFAs are transported across the blood–brain barrier into the central nervous system where they regulate growth and the differentiation of neurons and synapses and control neuropeptides that suppress appetite [12,13]. Finally, SCFAs contribute to the permeability of the blood–brain barrier; recolonization of germ-free mice with microbiota or SCFA-producing bacteria reconstitutes blood–brain barrier integrity, consistent with their protective role in MS [14].

SCFAs—primarily acetate, propionate and butyrate—account for 90% of SCFAs produced during the fermentation of indigestible carbohydrates and starch by the gut microbiota in the colon [15]. They regulate various physiological functions, including neuronal growth, energy homeostasis and immunity. Among these SCFAs, propionic acid has gained particular attention due to its anti-inflammatory properties [16,17,18]. It decreases the activity of pro-inflammatory T-helper cell responses (such as Th1 and Th17) while promoting regulatory T-cell activity, and thereby contributing to a balanced immune response [19]. This balance is crucial in controlling the overactive immune responses seen in autoimmune diseases like MS.

Additionally, propionic acid regulates metabolic functions, like production in adipose tissue adipokines, signaling molecules involved in energy metabolism. By promoting the oxidation of fatty acids, propionic acid reduces fatty acid levels in the liver. Furthermore, propionate produced from of microbial digestion of carbohydrates is a principal substrate of gluconeogenesis in the liver, which constitutes the major source of glucose for the body [20]. Thus, the dual roles of propionate in immune modulation and metabolic regulation suggest it may have therapeutic benefits, particularly in conditions like MS where both immune and metabolic dysregulation are implicated.

SCFAs exert their physiological effects by binding to specific SCFA-sensing G protein-coupled receptors, particularly rhodopsin-like G protein-coupled receptors 41 and 43 (GPCR41, GPR43). These receptors are expressed on various cell types, including immune cells, epithelium and endocrine cells, and are important regulators of metabolism and immunity. In addition, SCFAs also inhibit histone deacetylases (HDACs), influencing gene expression and further contributing to immune regulation.

Upon activation by SCFAs like propionic acid, GPRs expressed on pancreatic and enteroendocrine cells and adipocytes trigger the release of glucagon-like peptides (GPL-1 and GPL-2), which regulate energy homeostasis, insulin secretion and glucose metabolism. Their activation by propionic acid also stimulates leptin secretion from adipocytes. Additionally, they also activate the peroxisome proliferator-activated receptor gamma (PPAR-gamma) pathway and inhibit the nuclear factor-kappa B (NF-kB), a key effector in inflammation [4,15,21].

Gut bacteria directly contribute to energy absorption by providing energy substrates like propionate to the host [22,23]. These energy resources participate in energy homeostatic metabolism energy, entering host metabolism pathways including gluconeogenesis and lipogenesis. By contrast, the Western diet is enriched with health-damaging long-chain fatty acids (LCFAs).

Given the importance of gut bacteria, this review explores the pathophysiology of MS, emphasizing the influence of diet with a particular focus on propionic acid. It discusses the physiological functions of propionic acid; its production by the gut microbiome; its impact on the immune system; and the potential benefits of supplementation. The central idea is that propionic acid from the diet and from its production by gut microbiota is metabolized by gut microbiota while at the same time it regulates the composition of gut ecosystem microbiota. Both effects influence systemic immune responses as “immunometabolism decreasing autoimmunity”.

Here, we review the evidence for the protective role of propionic acid in the pathophysiology of MS, featuring its impact on the immune system and the gut microbiome, and discuss the possibilities of supportive dietary supplementation. Its potential as a dietary supplement to support MS management is intriguing, as it could provide a natural way to enhance immune regulation and reduce inflammation also through diet.

## 2. Propionate Function

**Figure 1:**
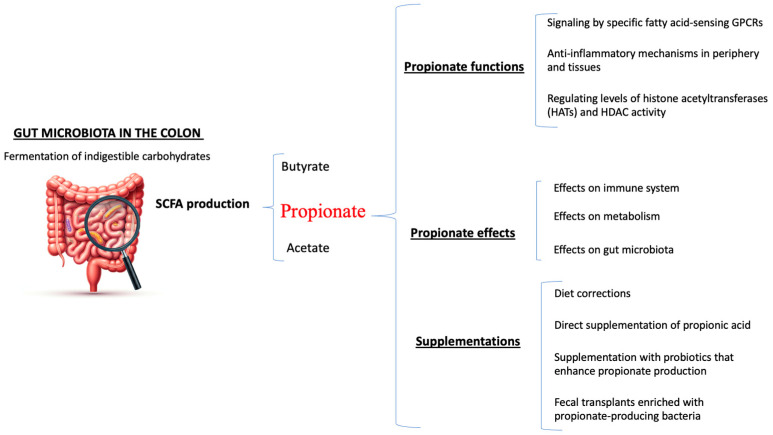
Schematic representation of propionate’s functions, effects and supplementation in MS.

Propionate is a short chain fatty acid (SCFA). Propionate functions through three principal mechanisms: (i) regulating levels of histone acetyltransferases (HATs) and HDAC activity; (ii) signaling, by specific fatty acid-sensing GPCRs; and (iii) anti-inflammatory mechanisms in periphery and tissues (Figure 1) [11]. Propionate signaling includes surface-localized acid receptors or G protein-coupled receptors (GPCRs) expressed on epithelial cells, adipose tissue, and immune cells, including neutrophils, dendritic cells, macrophages and lymphocytes [19]. GPCRs sense dietary metabolites, and their activation suppresses cAMP-dependent signaling and activates alternative pathways including mTOR.

In the intestine, via GPR-41cAMP, propionate promotes gluconeogenesis, thereby controlling glucose homeostasis [24]. Through GPR-43, propionate also stimulates the production of gut hormones that reduce food intake and thus disfavor diet-induced obesity [25].

In white adipose tissue, propionate, via the GPR-43 (free fatty acid receptor 43, FFAR2)–PPAR-gamma2 pathway, demonstrates anti-lipolytic activity in vitro, reducing free fatty acids in vivo and promoting adipocyte differentiation in vitro [26,27]. Additionally, GPCR-41 (free fatty acid receptor 3, FFAR3) is highly expressed in the sympathetic nervous system, which is thus activated by propionate.

Finally, SCFAs modify the host epigenome (DNA methylation and histone modification) [28,29]. Specifically, propionic acid functions as an inhibitor of histone deacetylases (HDAC), a process that is independent of cell-surface receptors. This inhibition increases histone acetylation, leading to the opening of chromatin structures and upregulation of anti-inflammatory genes. For example, inhibition of HDAC by propionate enhances extrathymic production of regulatory T cells in the periphery [30].

Propionate induces a specifically GPCR43-dependent decrease in expression of HDAC6 and HDAC9 on colonic regulatory T cells [31].

Thus, propionate potentially has multiple epigenetic effects on MS via changes in energy metabolism; contributes to protection from obesity that increases risk of the disease; and regulates of histone modification and upregulation of anti-inflammatory genes.

## 3. Effects of Propionate Levels on MS

Several reports have found low levels of propionate in the feces and plasma of pwMS patients independently of their disease course and ethnic origin [17,32,33,34,35].

Gut dysbiosis, and thus deficiency in SCFAs, including propionate, were suggested to contribute to its initiation and progression [32]. The structural and functional changes in gut microbiome and related reductions in butyrate and propionate biosynthesis were also associated with the clinical severity of pwMS [33,35]. Furthermore, the levels of major SCFA significantly decrease in the blood of long-term active secondary progressive MS (SPMS) patients, associated with disease progression [34].

Propionate has multifocal effects on MS, including effects on the gut microbiome, immune system and central nervous system. The lower propionate levels in serum and feces of individuals with MS was more accentuated after a first relapse, suggesting that low propionate levels can be associated with worsening disease activity [17]. On the other hand, pwMS with higher propionate show fewer annual relapses, stabilization of disability, and reduced brain atrophy [17]. This finding indicates the possible therapeutic potential of propionate in modulating MS progression, possibly through the regulation of regulatory T cells, because a functional analysis of gut microbiomes from these patients revealed an increased expression of regulatory T cell-inducing genes in the intestine [17]. The same study showed that these effects of propionate supplementation in pwMS on the immune system were associated with long-term clinical improvement.

During pregnancy and postpartum, women with MS who are at risk of relapses presented with a low propionate/acetate ratio in the first trimester of gestation [36]. This imbalance in the SCFA ratio could predict an increased risk of relapses, especially postpartum, when major changes in immunity occur. Interestingly, the level of maternal propionate impacts embryo development. It has been observed in mice that propionate enters fetal circulation, controlling insulin levels and the development of the sympathetic nervous system through GPR41 signaling [37]. Conceivably, through this way, propionate produced by gut bacteria of the mother might also indirectly educate developing fetal immunity [38].

In a reverse-translational disease-in-a-dish model of human primary neurons, differentiated from MS patients’ induced pluripotent stem cells (iPSCs), the presence of propionic acid-induced neuroregeneration was discovered, leading to the recovery of damaged neurites [39]. This finding aligns with other studies showing propionate’s ability to promote neuroprotection by reducing oxidative stress and enhancing neuroregeneration in the peripheral nervous system, particularly in Schwann cells and dorsal root ganglia [40]. This effect is mediated by free fatty acid receptor 3 (FFAR3) in vitro and ex vivo along with induced histone acetylase expression [40].

The central role of the environment on MS etiopathology precludes the epigenetic modifications of different cells, including nervous and immune cells. In an EAE model, a subset of astrocytes accumulates an epigenetically determined immunological memory of past inflammation that causes exacerbated inflammation after rechallenge [41]. Thus, the increased levels of propionate acting through HDAC inhibition produce epigenetic remodulation of cells, counteracting the pro-inflammatory setting of autoimmunity [42].

Furthermore, the propionate could act on depression symptoms observed in pwMS. The propionate regulates neurotransmitter production with subsequent antidepressant effects [43]. However, that function is dose dependent, and it has been shown in animal models that low doses of propionate induce antidepressant effects, but high-doses may induce autism-like symptoms through induction of depletion of neurotransmitters. This study also underlined the cumulative toxicity of propionate as a food additive [44].

However, to date, evidence on how propionate levels influence MS susceptibility, manifestations, and progression is still limited.

## 4. Effects of Propionate on Immunity in MS

Propionic acid is a strong modulator of the systemic immune response, exerting its effect through the nutrition and bacterial metabolites on the systemic immune response. Indeed, dietary SCFAs are non-infectious triggers of gut mucosal immunity and affect T cell differentiation in the host gut [45]. In contrast, medium-chain and long-chain fatty acids polarize Th1 and Th17 cell differentiation in the small intestine and impair their intestinal sequestration through the p38-MAPK pathway [18]. These effects have been shown to aggravate experimental autoimmune encephalomyelitis (EAE) in the mouse model of MS, while SCFAs have an opposite effect, expanding beneficial regulatory T cells by suppressing the JNK1 and p38 pathways. SCFAs can thereby increase the levels of regulatory T cells and decrease pro-inflammatory Th1 and Th17 in periphery. This regulation occurs through activation of GPRs and/or the inhibition of histone deacetylases [18,46,47].

In the mouse model of MS with a high-fat-diet-induced immune response, propionate treatment prevented disease inhibiting Th17 mediated inflammation in the gut and the spleen and enhanced regulatory T cell levels and function trough p38-MAPK and IL-10 signaling [48]. These observations are further supported by clinical findings in obese pwMS, where reduced fecal propionate levels were associated with increased Th17 levels and decreased regulatory T cells compared to non-obese pwMS [48]. Additionally, SCFA-producing commensal *Bacteroides fragilis*, known to be protective in EAE, induces IL-10—resulting in the production of FoxP3+ regulatory T cells [49].

Propionate directly suppresses IL-17 production by gamma–delta T cells through an HDAC-dependent mechanism [50]. Independent studies have also observed a significant decrease in serum propionate levels in pwMS with clinically isolated syndrome (CIS) or MS compared to healthy controls. In this study, the levels of circulating T follicular regulatory cells and T follicular helper cells CD4+CXCR5+ were positively correlated with serum levels of propionate [35].

In the myeloid compartment, propionate inhibits maturation of monocyte-derived DCs induced by LPS in vitro, reducing CD8+T cell activation [51]. Propionate downregulates the expression of genes encoding cytokines and chemokines in both immature and mature myeloid dendritic cells [51]. Furthermore, SCFAs, including propionate, can promote the anti-inflammatory activity of antigen-presenting cells and T cells [34].

In the B cell compartment, propionate inhibits class-switch recombination, somatic hypermutation, and plasma cell differentiation in C57BL/J6 mice, thereby contributing to the suppression of autoimmune responses [52].

## 5. Propionic Acid and Gut Microbiota in MS

Many studies have examined shifts in microbiome and related changes in propionic acid levels in a variety of experimental systems. The gut–brain axis involved in microbiota communication includes the immune, hypothalamic–pituitary–adrenal axis, serotonin/tryptophan/kynurenine, vagal, neuroendocrine and metabolome signaling [53,54]. In general, the composition of the microbiome is primarily affected by numerous factors including an individual’s body mass index (BMI), fasting glucose levels, glycemic status, high density lipoproteins (HDL), cholesterol, waist circumference, hip circumference and lactose consumption [55]. Accordingly, the composition of the gut microbiota can determine the efficiency of energy production from food, and thus, changes in diet have been associated with modifications in the microbial ecosystem in the gut [4].

It is conceivable that variations in the metabolome correlate with microbiome composition. In particular, reduced levels of propionate were associated with a reduction in SCFA-producing bacteria, a hallmark of the gut microbiota in MS [56].

Diverse bacteria produce propionate: Gram-positive bacteria (Bacteroides) principally produce acetic acid and propionic acid, while Gram-positive bacteria (Firmicutes) produce principally butyric acid. The main producers of propionic acid are anaerobic bacteria, including *Propionibacteria*, *Veillonella*, *Selenomonas* and *Clostridium* including *Clostridium propionicum*.

It has been shown that strains exposed to propionic acid outcompete wild-type strains in a humanized murine model [57].

Although reduced microbial gene richness is associated with dysmetabolism and sub-inflammation, microbiota diversity in MS remains relatively stable. However, changes in the abundance of certain bacterial taxa have been observed in MS, including decreases in SCFA-producing bacteria like *Butyrycimonas*. Predominant in gut SCFA-production, *Butyricimonas* abundance correlates negatively with the expression of pro-inflammatory genes in T cells and monocytes in pwMS [8].

As another example, dietary regimens as like intermittent fasting and fasting-mimicking increases the abundance of *Akkermansia muciniphila*, a synthesizer of propionate [58,59].

Several bacterial groups for which abundance is decreased in pwMS—like *Clostridium*, *Faecalobacterium*, *Eubacterium*, *Ruminococcus*, *Butyricimonas*, *Bacteroidetes* and *Prevotella*—participate in SCFA metabolism [60,61,62]. Of particular interest are *Clostridium* (Firmicutes), which are prevalent gut commensals strongly associated with SCFA production and greatly affecting the maintenance of gut homeostasis [63]. The immunosuppressive function of *Clostridium* is mediated through the induction of regulatory T cells and includes SCFA production [64].

The Clostridia include anti-inflammatory bacteria such as like *Eubacterium*, *Ruminococcus* and *Faecalibacterium* [61]. However, interferon-beta treatment of MS was associated with dysbiosis of SCFA-producing bacteria like *Ruminococcus* sp., *Clostridium* sp., *Faecalibacterium prausnitzii*, and *Roseburia inulinivorans*.

In other dietary effects, an excess of long acids reduces the abundance of *Prevotellaceae* and *Bacteroidetes*. Conversely, a fiber-rich diet promotes the fermentation process, increasing the number of *Bacteroidetes*, which are involved in SCFA production as indicated above. Interestingly, and in parallel with the recently observed impact of obesity on MS, depletion of *Bacteroidetes* in those cases has also been observed, linking obesity with the disease’s pathogenesis [18]. In a small randomized controlled trial, six weeks of supplementation with inulin-type fructans that had a bifidogenic and a *Bacteroidetes* effect led to increased concentrations of total fecal SCFAs (propionic and acetic) without changing the diversity of the fecal microbial ecosystem [65].

Recently, it has been shown that gut microbiota could metabolize phytate, considered until now as antinutritional in humans, to propionate. Phytate is a major form of phosphate in plant seeds and grains that also functions as a strong chelator of mineral ions. In particular, *Mitsuokella jalaludinii*—which degrades phytate in synergy with *Anaerostipes rhamnosivorans*—produces the SCFA propionate. Both species are prevalent in the human gut and can contribute to phytate-driven health benefits [66].

In a trophic chain of phytate metabolism, *Mitsuokella* spp. is an efficient and prevalent phytate-degrading bacteria whose abundance is strongly associated with ethnic origin. *M. jalaludinii* produces anti-microbial 3-hydroxypropionate that can be converted by *Anaerostipes rhamnosivorans* to propionate that improve epithelial barrier integrity [66]. 3-hydroxypropionate is known to be anti-microbial [67]. These instances point toward the interaction of commensal bacteria in the human colon.

Supplementation of *A. rhamnosivorans* in fecal phytate in Western-diet-fed mice increases production of propionate [68].

Furthermore, *Lentilactobacillus* spp., including *Limosilactobacillus reuteri,* commonly used as probiotic in antibiotic therapy, are the producers of reuterin, which is metabolized to antibacterial 3-hydroxypropionic acid [67]. This points to feeding competition between gut microbiota that could change in turn its composition.

A multi-OMICS study integrating diet composition, immune responses, blood metabolomics and microbiome composition identified multisystem alterations in MS. Interestingly, meat intake in diet contributed to a reduction in SCFA levels, a decrease in *Bacteroides thetaiotaomicron* and a parallel increase in Th17 cell levels [69]. Thus, it increased inflammation.

Recently, it has been shown that a dietary prebiotic, ellagic acid (a natural polyphenol rich in the Mediterranean diet) effectively stopped the progression of EAE, through the regulation of the microbiota–metabolite–immunity axis. This prebiotic increased the abundance of SCFA-producing bacteria such as *Alloprevotella*, *Bacteroides* and *Prevotella* [70].

On the other hand, the expanding of *Firmicutes* is characteristic of MS and leads to endotoxemia, and thus to chronic inflammation contributing to the disease [71,72].

The other strong factor changing gut microbiota is antibiotic use, and it has been shown that treatment with vancomycin or broad-spectrum antibiotics that target Gram-positive bacteria cause drastic reduction in the production of SCFAs by gut microbiota [50].

In conclusion, accumulating evidence suggests that reductions in SCFA-producing bacteria, including propionate, contribute to pro-inflammatory states in pwMS.

## 6. Propionate Strengthens Permeability of Natural Barriers

The interactions between gut-derived microbial metabolites and the blood–brain barrier (BBB) play a central role of the gut–brain axis. Preclinical studies have shown that microbial metabolites can influence barrier functioning, linking gut microbiota alterations to barrier dysfunction and following the abnormal passage of microbial-derived and non-microbial molecules through the gut–brain axis. Barrier dysfunction is a hallmark of disorders affecting the brain and the gut, as could be proposed to define MS, and may explain some of the comorbidities observed in these conditions [73]. An imbalance in gut microbiota affects natural barriers, including the BBB, the blood–cerebrospinal fluid barrier, and the gut, vascular and intestinal epithelial barrier [74,75]. Increased permeability of these barriers is characteristic of neurodegenerative diseases and neuroinflammation; thus, the study of microbiota-derived signaling molecules, such as SCFAs like propionate, is critical for exploring potential therapies for the homeostasis of barrier integrity [76]. Microbial metabolites, including SCFA, have shown promise in protecting the BBB [77]. Specifically, SCFAs have been demonstrated to protect tight junction protein expression [14,78].

Propionate functions in maintenance of natural defensive barriers in humans, although its precise mechanism of function is not fully understood. Specifically, butyrate and propionate enhance remodeling of the actin cytoskeleton and tight junction protein (such as zonula occludens-1 and claudin 5) and regulate their interactions [79]. It has been demonstrated in vitro that propionate strengthens the BBB through the propionate receptor FFAR3 on the human brain endothelium, and protects the barrier from microbial infections and oxidative stress [78]. It has been shown, in different human brain and colonic cell lines, that SCFAs, including propionate, also protect from LPS-mediated morphological disruption of zonula occludens and claudin proteins and from LPS-mediated barrier disruption through NOD-, LRR- and pyrin domain-containing protein 3 (NLPR3) inflammasome and inhibition of autophagy [78,79,80]. Also, propionate strengthens tight junction barriers in vitro in human intestinal Caco-2 cells [81].

Future studies will identify specific bacterial strains and metabolites produced by them, including propionate, to better understand their roles in the homeostasis and regulation of BBB function.

## 7. Propionic Acid Supplementation in MS

In general, dietary approaches focusing on whole foods rich in microbiota-accessible carbohydrates, such as fruits and vegetables, provide benefits for modulation of gut ecosystem microbiota. These foods promote the growth of anti-inflammatory *Bifidobacterium* and *Lactobacillus* while inhibiting detrimental *Escherichia coli* and *Enterococcus* spp. [82]. Fruits and vegetables are rich in microbiota-accessible carbohydrates like oligosaccharides, pectin, cellulose, inulin, lignans and starch. These are fermented by the gut microbiota in the large intestine to produce SCFAs: acetate, propionate, butyrate and lactate—and thus, link to the regulation of the bacterial ecosystem’s composition. Despite this knowledge, dietary strategies are underutilized in clinical practice, limiting their broader application in MS [82,83,84].

Increased levels of propionate can be achieved through various methods: (i) diet corrections; (ii) direct supplementation of propionic acid; (iii) supplementation with probiotics that enhance propionate production; and (iv) fecal transplants enriched with propionate-producing bacteria (Figure 1).

Although already present in small quantities in common diets, propionic acid can easily be supplemented and may prove beneficial for individuals with MS, including those undergoing immunotherapy. Propionic acid is commonly used as a fungistatic agent for bread and bakery products and can be produced by whey fermentation using strains of *Propionibacterium*. Additionally, nutritional supplements such as yeast extract also increase the production of propionate [85]. Furthermore, propionic acid fermentation is performed by different bacteria from the genus *Propionibacterium*, which colonize cheese during its maturation process [86].

The positive effects of propionate, such as a reduction in food intake, inhibition of lipolysis and decrease in plasma fatty acid levels, have been observed in a study when high concentrations of propionate were used (10-fold higher than those used for food preservation). Remarkably, three years of propionic acid supplementation in MS led to fewer annual relapses, had a stabilizing effect on disability, and reduced brain atrophy. However, this study focused on immune and neurological outcomes rather than anthropometric and metabolic measures [17]. Mice on a high-fat diet, rich in lauric acid, during EAE, exhibited more severe disease progression, including enhanced demyelination and immune cell infiltration in the spinal cord. These effects of a high-fat diet on EAE were prevented with propionic acid treatment, by inhibiting T helper 17-mediated inflammation in the gut and spleen. In a subsequent study of obese and non-obese pwMS, treatments with propionic acid for 90 days restored regulatory T cell–T helper 17 homeostasis [48].

Propionic acid supplementation has also been shown to be beneficial in patients with colitis, reducing disease severity and suggesting potentials for treatment of inflammatory bowel disease. These findings open the possibility of translating propionate supplementation to other autoimmune disorders, including MS.

In the EAE model, oral treatment with SCFAs or a high-fiber diet ameliorated symptoms and increased the levels of regulatory T cells in the draining nodes [87]. Supplementation can also be performed by enrichment of the taxa producing propionate with probiotic strains like *Lactobacillus* and *Bifidobacterium*, which produce propionate. For instance, *Lactobacillus plantarum* P-8 has been shown to reduce opportunistic pathogens, increase *Bifidobacterium* abundance and enhance production of propionate [88].

Notably, *Bacillus amyloliquefaciens*-supplemented camel milk prevented MS symptoms in an MOG immunized C57BL6J mice model, increasing levels of SCFAs including propionate [89].

Another approach includes fecal transplants enriched in propionic acid and propionate-producing bacteria, that ameliorated clinical symptoms of EAE [90].

In a small clinical study, supplementation with propionate was found to protect against osteoporosis co-morbidity in MS, increasing serum levels of osteocalcin—a marker of bone formation—and reducing beta-CrossLaps—a marker of bone resorption. The same study showed that the levels of regulatory T cells and their suppressive capacity positively correlated with osteocalcin, while Th17 levels were inversely correlated [91]. However, these results are preliminary, and supplementation protocols are currently only conducted in research settings.

## 8. Precision Microbiome Modulation with Dietary Fiber Interventions in MS

Dietary intervention is one of the most accessible supplementary approaches studied for MS and may: (i) directly provide missing nutrients; (ii) provide components that are metabolized by gut microbiota into the missing components; (iii) regulate the gut microbial ecosystem composition and function by enriching beneficial species that impact metabolism, the immune system and the nervous system. One promising therapeutic strategy for modulating the MS—associated microbiota and thereby influencing immune and metabolic status—is a high-fiber diet, the source of SCFAs, such as propionate [92]. A diet rich in non-digestible carbohydrates has been shown to increase peripheral propionate levels tenfold [93,94]. Conceivably, combining fiber-based prebiotics with probiotics enhances probiotic efficacy. Specific foods rich in starch increase both SCFA producers and SCFA production. As an example, tapioca starch increased the abundance of *Parabacteroides distasonis* and, specifically, propionate levels, in a dose dependent manner [95]. Similarly, green bananas boost SCFA level, including that of propionate, in feces [96].

Dietary fermentable and non-fermentable fiber prevent central nervous system autoimmunity through shifts in microbiota and subsequent metabolic and immune changes [97].

A high-fiber diet appears to benefit MS outcomes directly and indirectly. For example, shifts in the MS-associated microbiota may be explained by poor-in-fiber diets and a reduced abundance of propionate-producing bacteria [98]. Furthermore, *Prevotella*, a marker of long-term fiber intake and *Christensenellaceae*, associated with altered metabolism and reduced obesity, are less abundant in human MS and EAE [99]. Additionally, animals fed resistant grains show reduced abundance of *Methanobrevibacter* [100], a genus enriched in pwMS.

Observational study indicated that a high-plant diet correlates with a reduced risk of MS [101]. In pediatric MS patients, a diet rich in fiber was associated with a lower odds of disease, suggesting a protective effect potentially mediated by gut microbiota [102]. Moreover, the metagenome in pediatric onset of MS reflects diets low in fiber [103].

In a small study, a high-vegetable/low-protein diet, the improvement of clinical parameters in pwMS through the increase in anti-inflammatory responses guided by shifts in the microbiome [104]. A very-low-fat, plant-based diet also improved fatigue, BMI and metabolic biomarkers in pwMS [105].

Similarly, a Mediterranean diet rich in fiber reduced markers of intestinal barrier dysfunction, including plasma lipopolysaccharide-binding proteins and fecal zonulin, through the increased levels of propionate and butyrate [106].

Conversely, a large study of the MS microbiome found only modest associations between diet and microbiome composition and function, noting that while a healthier diet correlates with higher microbiome diversity, it is not the only factor regulating gut microbiota composition and function. Some bacteria remain unaffected by dietary changes due to preexisting microbiota and environmental factors [5]. Additionally, fiber’s impact on inflammation also varies by baseline inflammation levels [107], and fiber’s effect on inflammation markers was reduced in individuals with lower microbiota richness [4].

A high-fiber diet has to be considered within a broader context, as individual responses to dietary fiber differ. Some individuals benefit from simple dietary intervention, while others require additional intervention with probiotics to enhance the functional capacity of the microbiota. Additionally, the individuals with increased systemic inflammation on high-fiber diets also present with lower capacity to produce propionate [108]. Given these differences, future studies will likely employ microbial community-scale metabolic modeling to predict individualized SCFA production profiles and measure the different dietary, prebiotic and probiotic intake to design personalized dietary, prebiotic and probiotic interventions to optimize SCFA production in the gut [108].

Although research on dietary interventions for MS is expanding, there is no recommendation for a specific diet [109]. Large-scale clinical studies are underway to introduce dietary interventions in MS.

## 9. The Association of Obesity with MS—Immunometabolism of an Immune-Mediated Disorder

The effects of obesity on MS are multifaced, contributing to increased disease risk and progression through increases in inflammation, metabolic dysregulation and reduced physical fitness. The risk factors for MS include both environmental exposure and a genetic predisposition to obesity. Obesity, especially during childhood and adolescence, has been associated with an increased risk of MS [110,111,112,113]. Obese pwMS present more frequently with health comorbidities, such as increased incidence of brain lesions and atrophy, higher levels of disability, depression and an overall reduced quality of life [114,115,116]. A high percent of body fat and poor diet are frequently observed in adult pwMS, and improving lifestyle and diet has been recommended to reduce fatigue and improve quality of life [117]. Furthermore, obesity in newly diagnosed pwMS is associated with higher disease severity and poorer outcomes [118]. Additionally, polygenic obesity has been confirmed in MS by a Mendelian randomization study [119].

Propionate may potentially counteract obesity in MS by influencing both metabolism and immune systems. In overweight and obese individuals, the overstimulation of T lymphocytes by nutrients and energy-detecting pathways causes immunometabolic states that lead to the interruption of immunological self-tolerance contributing to autoimmunity [120].

Obesity is a recognized factor in inflammation and immune system dysregulation, promoting immunometabolism (metabolic reprogramming of immune cells). Adipocyte tissue contributes to the regulation of systemic immune response through adipocytokines, and this relationship is bidirectional, because immune cells also regulate adipocyte homeostasis and metabolism by releasing both pro-inflammatory and anti-inflammatory cytokines [121]. Both propionate and butyrate induce anorexigenic hormones and leptin [25,122]. The physiological daily adipocytokine leptin production that determines mTOR activity through feeding and fasting is disrupted in obesity because of continuous food consumption, although it is central for regulatory T cell expansion that suppresses pro-inflammatory T helper 1 and T helper 17 cells, and it also regulates of the activation and migration of neutrophils, macrophages and monocytes [120,123,124]. In MS, increased leptin levels promote autoreactive T cell proliferation and pro-inflammatory cytokine secretion and inhibited regulatory T cell proliferation [125,126]. In untreated obese pwMS, elevated leptin, and parallel increased levels of circulating nutrients, triggered inflammatory immune responses, like the conversion of conventional T cells into inflammatory T helper 1 and T helper 17 cells, which compromises immunological self-tolerance and dysregulation of T-cell receptor-mediated signaling through the mTOR pathway. This process downregulates the expression of the forkhead-box P3 (FOXP3) gene and, consequently, the CD4+CD25+FOXP3+ regulatory T cells [120,121,127]. Supporting these results, leptin can exacerbate EAE [128]. Propionate counteracts this process, inducing regulatory cells in the gut trough inhibition of histone deacetylases at the Foxp3 locus, and inducing its expression on regulatory T cells [129]. Interestingly, intermittent fasting in EAE and pwMS has been shown to enrich the intestinal microbiota with *Lactobacillaceae*, *Bacteridaceae* and *Prevotellaceae*, known SCFA-producers, which was associated with decreases in leptin levels, reduction in IL-17-producing T cells and increases in regulatory T cells [130].

It is widely known that SCFAs improve insulin secretion. Growth factors acting on nutrition—leptin, insulin and insulin-like growth factor 1 (IGF-1) activate mTOR signaling in immune cells, and thus regulate immunometabolism, and when impaired contribute to inflammation and autoimmunity. Different pro-inflammatory cells accumulate in adipose tissue and secrete pro-inflammatory cytokines—interleukin 1, tumor necrosis factor, IL-6, produced by pro-inflammatory macrophages, IL-17 and interferon-gamma—produced by a subset of CD4+T cells and leptin produced by adipocytes, contributing to tissue damage and thus accelerate autoimmunity [131]. Consequently, treatments that reduce leptin levels, like metformin, thioazolinediones, pioglitazone and statins, which are used to treat metabolic syndrome and insulin resistance, have also demonstrated benefits in downregulating inflammation in EAE and in pwMS with metabolic syndrome [132].

Future therapies will consist of combinatorial therapies that will include, in addition to immune-modifying therapies, immunometabolism downregulating interventions, including diet interventions with reduced food intake or caloric restriction and supplementation for missing components as like propionate and probiotics.

## 10. Limitations and Challenges

In spite of the promising preliminary findings on the benefits of propionate supplementation in humans, some studies have linked propionate oral supplementation to negative outcomes, such as high BMIs, insulin resistance and glucose intolerance, with limited benefits compared to propionate delivered by bacteria in the colon [133].

SCFAs induce a tolerogenic and immunosuppressive phenotype in the gut mucosa, but they have also been shown to potentiate a pro-inflammatory response. This highlights the need for dose-dependent studies to better understand their effects.

Additionally, propionic acid supplementation has to be undertaken with care, in view of a recent study that describes its propensity to promote the virulent phenotype of Crohn’s disease, associated with a higher abundance of *Enterobacteriaceae* and, in particular, adherent-invasive *Escherichia coli* [57]. This underscores the importance of microbiota screening during propionate supplementation to detect the potential for enrichment of harmful taxa.

Especially because the immune basal state differs among individuals in the general population, larger studies are required to assess the impact of propionate on MS more accurately. OMIC studies can estimate the global metabolic state and microbiome interactions and changes, while also accounting for factors such as diet and BMI on the results [134]. Additionally, the effects of placebos have to be considered, and replication studies are necessary to validate findings.

The impact and interaction of propionate on disease-modifying therapies used in MS remains unknown and requires further investigation. And, further research is also necessary to determine the effects of MS immunotherapies on the gut microbiome [135].

As for future directions, the effects of SCFAs on HAT- and HDAC-mediated post-translational modification of transcription factors could be explored; epigenetic regulation could also be examined in detail. Understanding these mechanisms could lead to the development of new therapies for neurodegenerative diseases and behavioral disorders, including effects relating to the strengthening of blood barrier integrity, modulating neurotransmission and influencing levels of neurotropic factors [11].

An alternative to direct supplementation with propionate would be changes in diet. The gut microbiome rapidly responds to a changed diet; a whole grain diet increases the propionic acid levels in plasma, while restriction of dietary fiber leads to a higher pH in the large intestine. Interventions in the diet rather than direct supplementation of propionate could thus be more effective because of the long-lasting effects accompanied by the expansion of probiotics that produce SCFAs.

However, more studies are needed on the characterization of MS-associated microbiome profiles to identify specific beneficial probiotics because, for example, supplementation with *Lactiplantibacillus plantarum* YRL45 [136] upregulates the abundance of propionic acid-producer *Akkermansia* bacteria that currently have a controversial function in MS. It will take considerable study to decide whether a diet rich in missing compounds is safer than direct propionic acid substitution.

## 11. Conclusions and Prospects

Metabolic impairment has been described for MS, and recent mechanistic studies of the metabolic pathways contributing to the disease pathophysiology are beginning to identify novel targets for therapeutic interventions. In line with the decreased abundance of SCFA-producing bacteria in MS, propionic acid is significantly reduced in the blood and stool of pwMS, particularly after relapse. Supplementation with propionic acid has been shown to improve regulatory T cell function, and long-term supplementary therapy reduced relapse, disability and brain atrophy. The severity of MS could have increased during human evolution as fiber consumption, the source of propionate, has decreased significantly over time, as human dietary habits have changed drastically [11].

Multiomics approaches are key to advancing personalized interventions, considering the high heterogeneity of the disease’s presentation and course. Simply, changes in diet could have an impact on both supplementation of missing components as propionate and the composition of gut microbiome. Additionally, manipulation of the gut microbiome to promote SCFA-producing bacteria could supplement missing propionate. However, larger clinical trials with a focus on reducing clinical pwMSs’ heterogeneity and controlling for placebo effects are needed to confirm these findings.

## References

[1] Reich D.S., Lucchinetti C.F., Calabresi P.A. (2018). Multiple Sclerosis. N. Engl. J. Med..

[2] Bhargava P., Fitzgerald K.C., Calabresi P.A., Mowry E.M. (2017). Metabolic Alterations in Multiple Sclerosis and the Impact of Vitamin D Supplementation. JCI Insight.

[3] Hedström A.K., Brenner N., Butt J., Hillert J., Waterboer T., Olsson T., Alfredsson L. (2021). Overweight/Obesity in Young Adulthood Interacts with Aspects of EBV Infection in MS Etiology. Neurol. Neuroimmunol. Neuroinflamm.

[4] Cotillard A., Kennedy S.P., Kong L.C., Prifti E., Pons N., Le Chatelier E., Almeida M., Quinquis B., Levenez F., Galleron N. (2013). Dietary Intervention Impact on Gut Microbial Gene Richness. Nature.

[5] iMSMS Consortium (2022). Gut Microbiome of Multiple Sclerosis Patients and Paired Household Healthy Controls Reveal Associations with Disease Risk and Course. Cell.

[6] Berer K., Gerdes L.A., Cekanaviciute E., Jia X., Xiao L., Xia Z., Liu C., Klotz L., Stauffer U., Baranzini S.E. (2017). Gut Microbiota from Multiple Sclerosis Patients Enables Spontaneous Autoimmune Encephalomyelitis in Mice. Proc. Natl. Acad. Sci. USA.

[7] Cekanaviciute E., Yoo B.B., Runia T.F., Debelius J.W., Singh S., Nelson C.A., Kanner R., Bencosme Y., Lee Y.K., Hauser S.L. (2017). Gut Bacteria from Multiple Sclerosis Patients Modulate Human T Cells and Exacerbate Symptoms in Mouse Models. Proc. Natl. Acad. Sci. USA.

[8] Jangi S., Gandhi R., Cox L.M., Li N., von Glehn F., Yan R., Patel B., Mazzola M.A., Liu S., Glanz B.L. (2016). Alterations of the Human Gut Microbiome in Multiple Sclerosis. Nat. Commun..

[9] Chesné J., Cardoso V., Veiga-Fernandes H. (2019). Neuro-Immune Regulation of Mucosal Physiology. Mucosal Immunol..

[10] Silva Y.P., Bernardi A., Frozza R.L. (2020). The Role of Short-Chain Fatty Acids From Gut Microbiota in Gut-Brain Communication. Front. Endocrinol..

[11] van der Hee B., Wells J.M. (2021). Microbial Regulation of Host Physiology by Short-Chain Fatty Acids. Trends Microbiol..

[12] Vijay N., Morris M.E. (2014). Role of Monocarboxylate Transporters in Drug Delivery to the Brain. Curr. Pharm. Des..

[13] Bachmann C., Colombo J.P., Berüter J. (1979). Short Chain Fatty Acids in Plasma and Brain: Quantitative Determination by Gas Chromatography. Clin. Chim. Acta.

[14] Braniste V., Al-Asmakh M., Kowal C., Anuar F., Abbaspour A., Tóth M., Korecka A., Bakocevic N., Ng L.G., Kundu P. (2014). The Gut Microbiota Influences Blood-Brain Barrier Permeability in Mice. Sci. Transl. Med..

[15] Al-Lahham S.H., Peppelenbosch M.P., Roelofsen H., Vonk R.J., Venema K. (2010). Biological Effects of Propionic Acid in Humans; Metabolism, Potential Applications and Underlying Mechanisms. Biochim. Biophys. Acta.

[16] Rothhammer V., Borucki D.M., Tjon E.C., Takenaka M.C., Chao C.-C., Ardura-Fabregat A., de Lima K.A., Gutiérrez-Vázquez C., Hewson P., Staszewski O. (2018). Microglial Control of Astrocytes in Response to Microbial Metabolites. Nature.

[17] Duscha A., Gisevius B., Hirschberg S., Yissachar N., Stangl G.I., Eilers E., Bader V., Haase S., Kaisler J., David C. (2020). Propionic Acid Shapes the Multiple Sclerosis Disease Course by an Immunomodulatory Mechanism. Cell.

[18] Haghikia A., Jörg S., Duscha A., Berg J., Manzel A., Waschbisch A., Hammer A., Lee D.-H., May C., Wilck N. (2015). Dietary Fatty Acids Directly Impact Central Nervous System Autoimmunity via the Small Intestine. Immunity.

[19] Mann E.R., Lam Y.K., Uhlig H.H. (2024). Short-Chain Fatty Acids: Linking Diet, the Microbiome and Immunity. Nat. Rev. Immunol..

[20] Engelking L.R. (2015). Textbook of Veterinary Physiological Chemistry.

[21] Lavelle A., Sokol H. (2020). Gut Microbiota-Derived Metabolites as Key Actors in Inflammatory Bowel Disease. Nat. Rev. Gastroenterol. Hepatol..

[22] Cani P.D., Van Hul M., Lefort C., Depommier C., Rastelli M., Everard A. (2019). Microbial Regulation of Organismal Energy Homeostasis. Nat. Metab..

[23] Wichmann A., Allahyar A., Greiner T.U., Plovier H., Lundén G.Ö., Larsson T., Drucker D.J., Delzenne N.M., Cani P.D., Bäckhed F. (2013). Microbial Modulation of Energy Availability in the Colon Regulates Intestinal Transit. Cell Host Microbe.

[24] De Vadder F., Kovatcheva-Datchary P., Goncalves D., Vinera J., Zitoun C., Duchampt A., Bäckhed F., Mithieux G. (2014). Microbiota-Generated Metabolites Promote Metabolic Benefits via Gut-Brain Neural Circuits. Cell.

[25] Lin H.V., Frassetto A., Kowalik E.J., Nawrocki A.R., Lu M.M., Kosinski J.R., Hubert J.A., Szeto D., Yao X., Forrest G. (2012). Butyrate and Propionate Protect against Diet-Induced Obesity and Regulate Gut Hormones via Free Fatty Acid Receptor 3-Independent Mechanisms. PLoS ONE.

[26] Hong Y.-H., Nishimura Y., Hishikawa D., Tsuzuki H., Miyahara H., Gotoh C., Choi K.-C., Feng D.D., Chen C., Lee H.-G. (2005). Acetate and Propionate Short Chain Fatty Acids Stimulate Adipogenesis via GPCR43. Endocrinology.

[27] Ge H., Li X., Weiszmann J., Wang P., Baribault H., Chen J.-L., Tian H., Li Y. (2008). Activation of G Protein-Coupled Receptor 43 in Adipocytes Leads to Inhibition of Lipolysis and Suppression of Plasma Free Fatty Acids. Endocrinology.

[28] Lee K.K., Workman J.L. (2007). Histone Acetyltransferase Complexes: One Size Doesn’t Fit All. Nat. Rev. Mol. Cell Biol..

[29] Roth S.Y., Denu J.M., Allis C.D. (2001). Histone Acetyltransferases. Annu. Rev. Biochem..

[30] Arpaia N., Campbell C., Fan X., Dikiy S., van der Veeken J., deRoos P., Liu H., Cross J.R., Pfeffer K., Coffer P.J. (2013). Metabolites Produced by Commensal Bacteria Promote Peripheral Regulatory T-Cell Generation. Nature.

[31] Smith P.M., Howitt M.R., Panikov N., Michaud M., Gallini C.A., Bohlooly-Y M., Glickman J.N., Garrett W.S. (2013). The Microbial Metabolites, Short-Chain Fatty Acids, Regulate Colonic Treg Cell Homeostasis. Science.

[32] Zeng Q., Gong J., Liu X., Chen C., Sun X., Li H., Zhou Y., Cui C., Wang Y., Yang Y. (2019). Gut Dysbiosis and Lack of Short Chain Fatty Acids in a Chinese Cohort of Patients with Multiple Sclerosis. Neurochem. Int..

[33] Takewaki D., Suda W., Sato W., Takayasu L., Kumar N., Kimura K., Kaga N., Mizuno T., Miyake S., Hattori M. (2020). Alterations of the Gut Ecological and Functional Microenvironment in Different Stages of Multiple Sclerosis. Proc. Natl. Acad. Sci. USA.

[34] Park J., Wang Q., Wu Q., Mao-Draayer Y., Kim C.H. (2019). Bidirectional Regulatory Potentials of Short-Chain Fatty Acids and Their G-Protein-Coupled Receptors in Autoimmune Neuroinflammation. Sci. Rep..

[35] Trend S., Leffler J., Jones A.P., Cha L., Gorman S., Brown D.A., Breit S.N., Kermode A.G., French M.A., Ward N.C. (2021). Associations of Serum Short-Chain Fatty Acids with Circulating Immune Cells and Serum Biomarkers in Patients with Multiple Sclerosis. Sci. Rep..

[36] Cuello J.P., Martínez Ginés M.L., García Domínguez J.M., Tejeda-Velarde A., Lozano Ros A., Higueras Y., Meldaña Rivera A., Goicochea Briceño H., Garcia-Tizon S., de León-Luis J. (2022). Short-Chain Fatty Acids during Pregnancy in Multiple Sclerosis: A Prospective Cohort Study. Eur. J. Neurol..

[37] Kimura I., Miyamoto J., Ohue-Kitano R., Watanabe K., Yamada T., Onuki M., Aoki R., Isobe Y., Kashihara D., Inoue D. (2020). Maternal Gut Microbiota in Pregnancy Influences Offspring Metabolic Phenotype in Mice. Science.

[38] Nyangahu D.D., Jaspan H.B. (2019). Influence of Maternal Microbiota during Pregnancy on Infant Immunity. Clin. Exp. Immunol..

[39] Gisevius B., Duscha A., Poschmann G., Stühler K., Motte J., Fisse A.L., Augustyniak S., Rehm A., Renk P., Böse C. (2024). Propionic Acid Promotes Neurite Recovery in Damaged Multiple Sclerosis Neurons. Brain Commun..

[40] Grüter T., Mohamad N., Rilke N., Blusch A., Sgodzai M., Demir S., Pedreiturria X., Lemhoefer K., Gisevius B., Haghikia A. (2023). Propionate Exerts Neuroprotective and Neuroregenerative Effects in the Peripheral Nervous System. Proc. Natl. Acad. Sci. USA.

[41] Lee H.-G., Rone J.M., Li Z., Akl C.F., Shin S.W., Lee J.-H., Flausino L.E., Pernin F., Chao C.-C., Kleemann K.L. (2024). Disease-Associated Astrocyte Epigenetic Memory Promotes CNS Pathology. Nature.

[42] Luu M., Pautz S., Kohl V., Singh R., Romero R., Lucas S., Hofmann J., Raifer H., Vachharajani N., Carrascosa L.C. (2019). The Short-Chain Fatty Acid Pentanoate Suppresses Autoimmunity by Modulating the Metabolic-Epigenetic Crosstalk in Lymphocytes. Nat. Commun..

[43] Li J., Hou L., Wang C., Jia X., Qin X., Wu C. (2018). Short Term Intrarectal Administration of Sodium Propionate Induces Antidepressant-Like Effects in Rats Exposed to Chronic Unpredictable Mild Stress. Front. Psychiatry.

[44] Hao C., Gao Z., Liu X., Rong Z., Jia J., Kang K., Guo W., Li J. (2020). Intravenous Administration of Sodium Propionate Induces Antidepressant or Prodepressant Effect in a Dose Dependent Manner. Sci. Rep..

[45] Li J., Jia H., Cai X., Zhong H., Feng Q., Sunagawa S., Arumugam M., Kultima J.R., Prifti E., Nielsen T. (2014). An Integrated Catalog of Reference Genes in the Human Gut Microbiome. Nat. Biotechnol..

[46] Haase S., Haghikia A., Wilck N., Müller D.N., Linker R.A. (2018). Impacts of Microbiome Metabolites on Immune Regulation and Autoimmunity. Immunology.

[47] Berer K., Mues M., Koutrolos M., Rasbi Z.A., Boziki M., Johner C., Wekerle H., Krishnamoorthy G. (2011). Commensal Microbiota and Myelin Autoantigen Cooperate to Trigger Autoimmune Demyelination. Nature.

[48] Haase S., Mäurer J., Duscha A., Lee D.-H., Balogh A., Gold R., Müller D.N., Haghikia A., Linker R.A. (2021). Propionic Acid Rescues High-Fat Diet Enhanced Immunopathology in Autoimmunity via Effects on Th17 Responses. Front. Immunol..

[49] Ochoa-Repáraz J., Mielcarz D.W., Ditrio L.E., Burroughs A.R., Begum-Haque S., Dasgupta S., Kasper D.L., Kasper L.H. (2010). Central Nervous System Demyelinating Disease Protection by the Human Commensal Bacteroides Fragilis Depends on Polysaccharide A Expression. J. Immunol..

[50] Dupraz L., Magniez A., Rolhion N., Richard M.L., Da Costa G., Touch S., Mayeur C., Planchais J., Agus A., Danne C. (2021). Gut Microbiota-Derived Short-Chain Fatty Acids Regulate IL-17 Production by Mouse and Human Intestinal Γδ T Cells. Cell Rep..

[51] Nastasi C., Fredholm S., Willerslev-Olsen A., Hansen M., Bonefeld C.M., Geisler C., Andersen M.H., Ødum N., Woetmann A. (2017). Butyrate and Propionate Inhibit Antigen-Specific CD8+ T Cell Activation by Suppressing IL-12 Production by Antigen-Presenting Cells. Sci. Rep..

[52] Sanchez H.N., Moroney J.B., Gan H., Shen T., Im J.L., Li T., Taylor J.R., Zan H., Casali P. (2020). B Cell-Intrinsic Epigenetic Modulation of Antibody Responses by Dietary Fiber-Derived Short-Chain Fatty Acids. Nat. Commun..

[53] Cryan J.F., O’Riordan K.J., Cowan C.S.M., Sandhu K.V., Bastiaanssen T.F.S., Boehme M., Codagnone M.G., Cussotto S., Fulling C., Golubeva A.V. (2019). The Microbiota-Gut-Brain Axis. Physiol. Rev..

[54] Schneider E., Balasubramanian R., Ferri A., Cotter P.D., Clarke G., Cryan J.F. (2024). Fibre & Fermented Foods: Differential Effects on the Microbiota-Gut-Brain Axis. Proc. Nutr. Soc..

[55] Rothschild D., Weissbrod O., Barkan E., Kurilshikov A., Korem T., Zeevi D., Costea P.I., Godneva A., Kalka I.N., Bar N. (2018). Environment Dominates over Host Genetics in Shaping Human Gut Microbiota. Nature.

[56] Melbye P., Olsson A., Hansen T.H., Søndergaard H.B., Bang Oturai A. (2019). Short-Chain Fatty Acids and Gut Microbiota in Multiple Sclerosis. Acta Neurol. Scand..

[57] Ormsby M.J., Johnson S.A., Carpena N., Meikle L.M., Goldstone R.J., McIntosh A., Wessel H.M., Hulme H.E., McConnachie C.C., Connolly J.P.R. (2020). Propionic Acid Promotes the Virulent Phenotype of Crohn’s Disease-Associated Adherent-Invasive Escherichia Coli. Cell Rep..

[58] Rangan P., Choi I., Wei M., Navarrete G., Guen E., Brandhorst S., Enyati N., Pasia G., Maesincee D., Ocon V. (2019). Fasting-Mimicking Diet Modulates Microbiota and Promotes Intestinal Regeneration to Reduce Inflammatory Bowel Disease Pathology. Cell Rep..

[59] Su J., Braat H., Peppelenbosch M.P. (2021). Gut Microbiota-Derived Propionate Production May Explain Beneficial Effects of Intermittent Fasting in Experimental Colitis. J. Crohns Colitis.

[60] Ríos-Covián D., Ruas-Madiedo P., Margolles A., Gueimonde M., de Los Reyes-Gavilán C.G., Salazar N. (2016). Intestinal Short Chain Fatty Acids and Their Link with Diet and Human Health. Front. Microbiol..

[61] Morrison D.J., Preston T. (2016). Formation of Short Chain Fatty Acids by the Gut Microbiota and Their Impact on Human Metabolism. Gut Microbes.

[62] De Filippo C., Cavalieri D., Di Paola M., Ramazzotti M., Poullet J.B., Massart S., Collini S., Pieraccini G., Lionetti P. (2010). Impact of Diet in Shaping Gut Microbiota Revealed by a Comparative Study in Children from Europe and Rural Africa. Proc. Natl. Acad. Sci. USA.

[63] Lopetuso L.R., Scaldaferri F., Petito V., Gasbarrini A. (2013). Commensal Clostridia: Leading Players in the Maintenance of Gut Homeostasis. Gut Pathog..

[64] Nagano Y., Itoh K., Honda K. (2012). The Induction of Treg Cells by Gut-Indigenous Clostridium. Curr. Opin. Immunol..

[65] Birkeland E., Gharagozlian S., Birkeland K.I., Valeur J., Måge I., Rud I., Aas A.-M. (2020). Prebiotic Effect of Inulin-Type Fructans on Faecal Microbiota and Short-Chain Fatty Acids in Type 2 Diabetes: A Randomised Controlled Trial. Eur. J. Nutr..

[66] De Vos W.M., Nguyen Trung M., Davids M., Liu G., Rios-Morales M., Jessen H., Fiedler D., Nieuwdorp M., Bui T.P.N. (2024). Phytate Metabolism Is Mediated by Microbial Cross-Feeding in the Gut Microbiota. Nat. Microbiol..

[67] Liang N., Neužil-Bunešová V., Tejnecký V., Gänzle M., Schwab C. (2021). 3-Hydroxypropionic Acid Contributes to the Antibacterial Activity of Glycerol Metabolism by the Food Microbe Limosilactobacillus Reuteri. Food Microbiol..

[68] Bui T.P.N., Mannerås-Holm L., Puschmann R., Wu H., Troise A.D., Nijsse B., Boeren S., Bäckhed F., Fiedler D., deVos W.M. (2021). Conversion of Dietary Inositol into Propionate and Acetate by Commensal Anaerostipes Associates with Host Health. Nat. Commun..

[69] Cantoni C., Lin Q., Dorsett Y., Ghezzi L., Liu Z., Pan Y., Chen K., Han Y., Li Z., Xiao H. (2022). Alterations of Host-Gut Microbiome Interactions in Multiple Sclerosis. EBioMedicine.

[70] Han B., Shi L., Bao M.-Y., Yu F.-L., Zhang Y., Lu X.-Y., Wang Y., Li D.-X., Lin J.-C., Jia W. (2024). Dietary Ellagic Acid Therapy for CNS Autoimmunity: Targeting on Alloprevotella Rava and Propionate Metabolism. Microbiome.

[71] Maynard C.L., Elson C.O., Hatton R.D., Weaver C.T. (2012). Reciprocal Interactions of the Intestinal Microbiota and Immune System. Nature.

[72] Brown K., DeCoffe D., Molcan E., Gibson D.L. (2012). Diet-Induced Dysbiosis of the Intestinal Microbiota and the Effects on Immunity and Disease. Nutrients.

[73] Aburto M.R., Cryan J.F. (2024). Gastrointestinal and Brain Barriers: Unlocking Gates of Communication across the Microbiota-Gut-Brain Axis. Nat. Rev. Gastroenterol. Hepatol..

[74] Tang W., Zhu H., Feng Y., Guo R., Wan D. (2020). The Impact of Gut Microbiota Disorders on the Blood-Brain Barrier. Infect. Drug Resist..

[75] Zhou A., Yuan Y., Yang M., Huang Y., Li X., Li S., Yang S., Tang B. (2022). Crosstalk Between the Gut Microbiota and Epithelial Cells Under Physiological and Infectious Conditions. Front. Cell Infect. Microbiol..

[76] Knox E.G., Lynch C.M.K., Lee Y.S., O’Driscoll C.M., Clarke G., Cryan J.F., Aburto M.R. (2023). The Gut Microbiota Is Important for the Maintenance of Blood-Cerebrospinal Fluid Barrier Integrity. Eur. J. Neurosci..

[77] O’Riordan K.J., Collins M.K., Moloney G.M., Knox E.G., Aburto M.R., Fülling C., Morley S.J., Clarke G., Schellekens H., Cryan J.F. (2022). Short Chain Fatty Acids: Microbial Metabolites for Gut-Brain Axis Signalling. Mol. Cell Endocrinol..

[78] Hoyles L., Snelling T., Umlai U.-K., Nicholson J.K., Carding S.R., Glen R.C., McArthur S. (2018). Microbiome-Host Systems Interactions: Protective Effects of Propionate upon the Blood-Brain Barrier. Microbiome.

[79] Knox E.G., Aburto M.R., Tessier C., Nagpal J., Clarke G., O’Driscoll C.M., Cryan J.F. (2022). Microbial-Derived Metabolites Induce Actin Cytoskeletal Rearrangement and Protect Blood-Brain Barrier Function. iScience.

[80] Feng Y., Wang Y., Wang P., Huang Y., Wang F. (2018). Short-Chain Fatty Acids Manifest Stimulative and Protective Effects on Intestinal Barrier Function Through the Inhibition of NLRP3 Inflammasome and Autophagy. Cell Physiol. Biochem..

[81] Isayama K., Rini D.M., Yamamoto Y., Suzuki T. (2023). Propionate Regulates Tight Junction Barrier by Increasing Endothelial-Cell Selective Adhesion Molecule in Human Intestinal Caco-2 Cells. Exp. Cell Res..

[82] Han Y., Xiao H. (2020). Whole Food-Based Approaches to Modulating Gut Microbiota and Associated Diseases. Annu. Rev. Food Sci. Technol..

[83] Wekerle H. (2018). Brain Inflammatory Cascade Controlled by Gut-Derived Molecules. Nature.

[84] Zoledziewska M. (2019). The Gut Microbiota Perspective for Interventions in MS. Autoimmun. Rev..

[85] Caballero B. (2003). Encyclopedia of Food Sciences and Nutrition.

[86] Fath B. (2019). Encyclopedia of Ecology.

[87] Mizuno M., Noto D., Kaga N., Chiba A., Miyake S. (2017). The Dual Role of Short Fatty Acid Chains in the Pathogenesis of Autoimmune Disease Models. PLoS ONE.

[88] Wang L., Zhang J., Guo Z., Kwok L., Ma C., Zhang W., Lv Q., Huang W., Zhang H. (2014). Effect of Oral Consumption of Probiotic Lactobacillus Planatarum P-8 on Fecal Microbiota, SIgA, SCFAs, and TBAs of Adults of Different Ages. Nutrition.

[89] Ibrahim H.I.M., Sheikh A., Khalil H.E., Khalifa A. (2023). Bacillus Amyloliquifaciens-Supplemented Camel Milk Suppresses Neuroinflammation of Autoimmune Encephalomyelitis in a Mouse Model by Regulating Inflammatory Markers. Nutrients.

[90] Chitrala K.N., Guan H., Singh N.P., Busbee B., Gandy A., Mehrpouya-Bahrami P., Ganewatta M.S., Tang C., Chatterjee S., Nagarkatti P. (2017). CD44 Deletion Leading to Attenuation of Experimental Autoimmune Encephalomyelitis Results from Alterations in Gut Microbiome in Mice. Eur. J. Immunol..

[91] Duscha A., Hegelmaier T., Dürholz K., Desel C., Gold R., Zaiss M.M., Haghikia A. (2022). Propionic Acid Beneficially Modifies Osteoporosis Biomarkers in Patients with Multiple Sclerosis. Ther. Adv. Neurol. Disord..

[92] Correale J., Hohlfeld R., Baranzini S.E. (2022). The Role of the Gut Microbiota in Multiple Sclerosis. Nat. Rev. Neurol..

[93] Vogt J.A., Pencharz P.B., Wolever T.M.S. (2004). L-Rhamnose Increases Serum Propionate in Humans. Am. J. Clin. Nutr..

[94] Nilsson A.C., Östman E.M., Knudsen K.E.B., Holst J.J., Björck I.M.E. (2010). A Cereal-Based Evening Meal Rich in Indigestible Carbohydrates Increases Plasma Butyrate the next Morning. J. Nutr..

[95] Deehan E.C., Yang C., Perez-Muñoz M.E., Nguyen N.K., Cheng C.C., Triador L., Zhang Z., Bakal J.A., Walter J. (2020). Precision Microbiome Modulation with Discrete Dietary Fiber Structures Directs Short-Chain Fatty Acid Production. Cell Host Microbe.

[96] Rabbani G.H., Ahmed S., Hossain I., Islam R., Marni F., Akhtar M., Majid N. (2009). Green Banana Reduces Clinical Severity of Childhood Shigellosis: A Double-Blind, Randomized, Controlled Clinical Trial. Pediatr. Infect. Dis. J..

[97] Berer K., Martínez I., Walker A., Kunkel B., Schmitt-Kopplin P., Walter J., Krishnamoorthy G. (2018). Dietary Non-Fermentable Fiber Prevents Autoimmune Neurological Disease by Changing Gut Metabolic and Immune Status. Sci. Rep..

[98] Schepici G., Silvestro S., Bramanti P., Mazzon E. (2019). The Gut Microbiota in Multiple Sclerosis: An Overview of Clinical Trials. Cell Transplant..

[99] Wu G.D., Chen J., Hoffmann C., Bittinger K., Chen Y.-Y., Keilbaugh S.A., Bewtra M., Knights D., Walters W.A., Knight R. (2011). Linking Long-Term Dietary Patterns with Gut Microbial Enterotypes. Science.

[100] Walker A.W., Ince J., Duncan S.H., Webster L.M., Holtrop G., Ze X., Brown D., Stares M.D., Scott P., Bergerat A. (2011). Dominant and Diet-Responsive Groups of Bacteria within the Human Colonic Microbiota. ISME J..

[101] Jahromi S.R., Toghae M., Jahromi M.J.R., Aloosh M. (2012). Dietary Pattern and Risk of Multiple Sclerosis. Iran. J. Neurol..

[102] Mirza A.I., Zhu F., Knox N., Black L.J., Daly A., Bonner C., Van Domselaar G., Bernstein C.N., Marrie R.A., Hart J. (2024). Mediterranean Diet and Associations with the Gut Microbiota and Pediatric-Onset Multiple Sclerosis Using Trivariate Analysis. Commun. Med..

[103] Mirza A.I., Zhu F., Knox N., Forbes J.D., Van Domselaar G., Bernstein C.N., Graham M., Marrie R.A., Hart J., Yeh E.A. (2022). Metagenomic Analysis of the Pediatric-Onset Multiple Sclerosis Gut Microbiome. Neurology.

[104] Saresella M., Mendozzi L., Rossi V., Mazzali F., Piancone F., LaRosa F., Marventano I., Caputo D., Felis G.E., Clerici M. (2017). Immunological and Clinical Effect of Diet Modulation of the Gut Microbiome in Multiple Sclerosis Patients: A Pilot Study. Front. Immunol..

[105] Yadav V., Marracci G., Kim E., Spain R., Cameron M., Overs S., Riddehough A., Li D.K.B., McDougall J., Lovera J. (2016). Low-Fat, Plant-Based Diet in Multiple Sclerosis: A Randomized Controlled Trial. Mult. Scler. Relat. Disord..

[106] Constantinides M.G., Link V.M., Tamoutounour S., Wong A.C., Perez-Chaparro P.J., Han S.-J., Chen Y.E., Li K., Farhat S., Weckel A. (2019). MAIT Cells Are Imprinted by the Microbiota in Early Life and Promote Tissue Repair. Science.

[107] Wastyk H.C., Fragiadakis G.K., Perelman D., Dahan D., Merrill B.D., Yu F.B., Topf M., Gonzalez C.G., Van Treuren W., Han S. (2021). Gut-Microbiota-Targeted Diets Modulate Human Immune Status. Cell.

[108] Quinn-Bohmann N., Wilmanski T., Sarmiento K.R., Levy L., Lampe J.W., Gurry T., Rappaport N., Ostrem E.M., Venturelli O.S., Diener C. (2024). Microbial Community-Scale Metabolic Modelling Predicts Personalized Short-Chain Fatty Acid Production Profiles in the Human Gut. Nat. Microbiol..

[109] Lorefice L., Pitzalis M., Zoledziewska M. (2024). Intermittent and Periodic Fasting—Evidence and Perspectives in Multiple Sclerosis. Mult. Scler. Relat. Disord..

[110] Munger K.L., Chitnis T., Ascherio A. (2009). Body Size and Risk of MS in Two Cohorts of US Women. Neurology.

[111] Chitnis T., Graves J., Weinstock-Guttman B., Belman A., Olsen C., Misra M., Aaen G., Benson L., Candee M., Gorman M. (2016). Distinct Effects of Obesity and Puberty on Risk and Age at Onset of Pediatric MS. Ann. Clin. Transl. Neurol..

[112] Munger K.L., Bentzen J., Laursen B., Stenager E., Koch-Henriksen N., Sørensen T.I.A., Baker J.L. (2013). Childhood Body Mass Index and Multiple Sclerosis Risk: A Long-Term Cohort Study. Mult. Scler..

[113] Huppke B., Ellenberger D., Hummel H., Stark W., Röbl M., Gärtner J., Huppke P. (2019). Association of Obesity With Multiple Sclerosis Risk and Response to First-Line Disease Modifying Drugs in Children. JAMA Neurol..

[114] Marck C.H., Neate S.L., Taylor K.L., Weiland T.J., Jelinek G.A. (2016). Prevalence of Comorbidities, Overweight and Obesity in an International Sample of People with Multiple Sclerosis and Associations with Modifiable Lifestyle Factors. PLoS ONE.

[115] Mowry E.M., Azevedo C.J., McCulloch C.E., Okuda D.T., Lincoln R.R., Waubant E., Hauser S.L., Pelletier D. (2018). Body Mass Index, but Not Vitamin D Status, Is Associated with Brain Volume Change in MS. Neurology.

[116] Fisher C.J., Heinberg L.J., Lapin B., Aminian A., Sullivan A.B. (2018). Depressive Symptoms in Bariatric Surgery Patients with Multiple Sclerosis. Obes. Surg..

[117] Moazen M., Mousavi-Shirazi-Fard Z., Mazloom Z., Izadi S., Ghaseminasab-Parizi M. (2024). Anthropometric Indices, Nutrient Intakes and Health-Related Characteristics of Patients with Multiple Sclerosis: A Cross-Sectional Study. Nutr. Neurosci..

[118] Lutfullin I., Eveslage M., Bittner S., Antony G., Flaskamp M., Luessi F., Salmen A., Gisevius B., Klotz L., Korsukewitz C. (2023). Association of Obesity with Disease Outcome in Multiple Sclerosis. J. Neurol. Neurosurg. Psychiatry.

[119] Mokry L.E., Ross S., Timpson N.J., Sawcer S., Davey Smith G., Richards J.B. (2016). Obesity and Multiple Sclerosis: A Mendelian Randomization Study. PLoS Med..

[120] Matarese G. (2023). The Link between Obesity and Autoimmunity. Science.

[121] de Candia P., Prattichizzo F., Garavelli S., Alviggi C., La Cava A., Matarese G. (2021). The Pleiotropic Roles of Leptin in Metabolism, Immunity, and Cancer. J. Exp. Med..

[122] Xiong Y., Miyamoto N., Shibata K., Valasek M.A., Motoike T., Kedzierski R.M., Yanagisawa M. (2004). Short-Chain Fatty Acids Stimulate Leptin Production in Adipocytes through the G Protein-Coupled Receptor GPR41. Proc. Natl. Acad. Sci. USA.

[123] La Cava A., Matarese G. (2004). The Weight of Leptin in Immunity. Nat. Rev. Immunol..

[124] Lord G.M., Matarese G., Howard J.K., Baker R.J., Bloom S.R., Lechler R.I. (1998). Leptin Modulates the T-Cell Immune Response and Reverses Starvation-Induced Immunosuppression. Nature.

[125] Matarese G., Carrieri P.B., La Cava A., Perna F., Sanna V., De Rosa V., Aufiero D., Fontana S., Zappacosta S. (2005). Leptin Increase in Multiple Sclerosis Associates with Reduced Number of CD4(+)CD25+ Regulatory T Cells. Proc. Natl. Acad. Sci. USA.

[126] Matarese G., Procaccini C., De Rosa V. (2008). The Intricate Interface between Immune and Metabolic Regulation: A Role for Leptin in the Pathogenesis of Multiple Sclerosis?. J. Leukoc. Biol..

[127] de Candia P., Procaccini C., Russo C., Lepore M.T., Matarese G. (2022). Regulatory T Cells as Metabolic Sensors. Immunity.

[128] Ouyang S., Hsuchou H., Kastin A.J., Mishra P.K., Wang Y., Pan W. (2014). Leukocyte Infiltration into Spinal Cord of EAE Mice Is Attenuated by Removal of Endothelial Leptin Signaling. Brain Behav. Immun..

[129] Park J., Kim M., Kang S.G., Jannasch A.H., Cooper B., Patterson J., Kim C.H. (2015). Short-Chain Fatty Acids Induce Both Effector and Regulatory T Cells by Suppression of Histone Deacetylases and Regulation of the mTOR-S6K Pathway. Mucosal Immunol..

[130] Cignarella F., Cantoni C., Ghezzi L., Salter A., Dorsett Y., Chen L., Phillips D., Weinstock G.M., Fontana L., Cross A.H. (2018). Intermittent Fasting Confers Protection in CNS Autoimmunity by Altering the Gut Microbiota. Cell Metab..

[131] Matarese G., Carrieri P.B., Montella S., De Rosa V., La Cava A. (2010). Leptin as a Metabolic Link to Multiple Sclerosis. Nat. Rev. Neurol..

[132] Negrotto L., Farez M.F., Correale J. (2016). Immunologic Effects of Metformin and Pioglitazone Treatment on Metabolic Syndrome and Multiple Sclerosis. JAMA Neurol..

[133] Tirosh A., Calay E.S., Tuncman G., Claiborn K.C., Inouye K.E., Eguchi K., Alcala M., Rathaus M., Hollander K.S., Ron I. (2019). The Short-Chain Fatty Acid Propionate Increases Glucagon and FABP4 Production, Impairing Insulin Action in Mice and Humans. Sci. Transl. Med..

[134] Lorefice L., Pitzalis M., Murgia F., Fenu G., Atzori L., Cocco E. (2023). Omics Approaches to Understanding the Efficacy and Safety of Disease-Modifying Treatments in Multiple Sclerosis. Front. Genet..

[135] Pilotto S., Zoledziewska M., Fenu G., Cocco E., Lorefice L. (2023). Disease-Modifying Therapy for Multiple Sclerosis: Implications for Gut Microbiota. Mult. Scler. Relat. Disord..

[136] Bu Y., Liu Y., Zhang T., Liu Y., Zhang Z., Yi H. (2023). Bacteriocin-Producing Lactiplantibacillus Plantarum YRL45 Enhances Intestinal Immunity and Regulates Gut Microbiota in Mice. Nutrients.

